# Differential Superiority of Heavy Charged-Particle Irradiation to X-Rays: Studies on Biological Effectiveness and Side Effect Mechanisms in Multicellular Tumor and Normal Tissue Models

**DOI:** 10.3389/fonc.2016.00030

**Published:** 2016-02-25

**Authors:** Stefan Walenta, Wolfgang Mueller-Klieser

**Affiliations:** ^1^Institute of Pathophysiology, University Medical Center, University of Mainz, Mainz, Germany

**Keywords:** radiobiology, particle irradiation, oxygen enhancement ratio, relative biological effectiveness, organotypic tumor and mucosa cultures, mucositis, cell migration

## Abstract

This review is focused on the radiobiology of carbon ions compared to X-rays using multicellular models of tumors and normal mucosa. The first part summarizes basic radiobiological effects, as observed in cancer cells. The second, more clinically oriented part of the review, deals with radiation-induced cell migration and mucositis. Multicellular spheroids from V79 hamster cells were irradiated with X-rays or carbon ions under ambient or restricted oxygen supply conditions. Reliable oxygen enhancement ratios could be derived to be 2.9, 2.8, and 1.4 for irradiation with photons, ^12^C^+6^ in the plateau region, and ^12^C^+6^ in the Bragg peak, respectively. Similarly, a relative biological effectiveness of 4.3 and 2.1 for ambient pO_2_ and hypoxia was obtained, respectively. The high effectiveness of carbon ions was reflected by an enhanced accumulation of cells in G_2_/M and a dose-dependent massive induction of apoptosis. These data clearly show that heavy charged particles are more efficient in sterilizing tumor cells than conventional irradiation even under hypoxic conditions. Clinically relevant doses (3 Gy) of X-rays induced an increase in migratory activity of U87 but not of LN229 or HCT116 tumor cells. Such an increase in cell motility following irradiation *in situ* could be the source of recurrence. In contrast, carbon ion treatment was associated with a dose-dependent decrease in migration with all cell lines and under all conditions investigated. The radiation-induced loss of cell motility was correlated, in most cases, with corresponding changes in β_1_ integrin expression. The photon-induced increase in cell migration was paralleled by an elevated phosphorylation status of the epidermal growth factor receptor and AKT-ERK1/2 pathway. Such a hyperphosphorylation did not occur during ^12^C^+6^ irradiation under all conditions registered. Comparing the gene toxicity of X-rays with that of particles using the γH2AX technique in organotypic cultures of the oral mucosa, the superior effectiveness of heavy ions was confirmed by a twofold higher number of foci per nucleus. However, proinflammatory signs were similar for both treatment modalities, e.g., the activation of NFκB and the release of IL6 and IL8. The presence of peripheral blood mononuclear cell increased the radiation-induced release of the proinflammatory cytokines by factors of 2–3. Carbon ions are part of the cosmic radiation. Long-term exposure to such particles during extended space flights, as planned by international space agencies, may thus impose a medical and safety risk on the astronauts by a potential induction of mucositis. In summary, particle irradiation is superior to gamma-rays due to a higher radiobiological effectiveness, a reduced hypoxia-induced radioresistance, a multicellular radiosensitization, and the absence of a radiation-induced cell motility. However, the potential of inducing mucositis is similar for both radiation types.

## Introduction

This article summarizes data which we have acquired in close collaboration with a number of scientists at the Gesellschaft fuer Schwerionenforschung (GSI) Darmstadt, Germany, for more than one decade. At the beginning of this collaboration, little was known about the basic radiobiology of particle irradiation, although there was emerging evidence already at that time for the usefulness of a carbon ion radiotherapy in clinical oncology (1). Consequently, the ultimate goal of the interactive work at the GSI accelerator was to augment our knowledge on biological effects of heavy charged particles in malignant tumors and in healthy tissue in comparison to the effect of conventional X-rays under equivalent conditions.

In most of the experiments, a carbon-12 (^12^C^6+^) beam was applied in the scanning mode either in the extended Bragg peak or in the plateau region at 227 MeV/nucleon. Conventional X-rays served as a reference, and X-ray equivalent doses were derived for heavy charged particle irradiation. Since beam-time is highly cost-intensive and since there is a pronounced competition among scientists for the acquisition of beam-time, design and performance of experiments with heavy charged particles are subjected to practical limitations by the restricted availability of the particle beam. This reduces the number of experiments within a given time frame and consequently restricts statistical corroboration of findings by multiple approaches. This has to be kept in mind, when data from particle irradiation are compared with those from other assays.

All irradiation experiments were carried out on cultured cells using various tumor and normal cell models. Besides conventional single cell cultures, complex three-dimensional (3D) cell cultures were used; these included organotypic cultures of the human oral mucosa with or without immune cells, planar cell multilayers of WiDr colon adenocarcinoma cells or of SiHa cervix carcinoma cells, and multicellular spheroids (MCS) from V79 cells. With a few exceptions, irradiation was routinely performed under standardized cell culture conditions at 37°C and ambient (20% O_2_) or reduced (pO_2_ close to 0 mmHg) oxygen supply conditions.

“Classical” radiobiological endpoints, such as clonogenic cell survival, spheroid volume growth, or cell cycle effects as a function of radiation dose, were used to quantify the efficiency of particle versus conventional radiation. Furthermore, the relative biological effectiveness (RBE) and the oxygen enhancement ratio (OER) for the two radiation modalities were derived. One specific endpoint was cell migration and motility in 2D and 3D conditions under the impact of irradiation. The gene toxicity of both radiation types in normal tissue was quantified using the γH2AX technique in organotypic mucosa cultures. In this multicellular model, assays for early events of a radiation-induced mucositis, such as activation of the transcription factor NFκB or release of cytokines IL6 and IL8, were applied. The cocultivation of the mucosa model with human peripheral blood mononuclear cells (PBMCs) revealed a significant role of immune cells in the emergence of radiation-related mucositis.

Following the introduction, the experimental part of this review article is subdivided into three major chapters. The first chapter deals with our data related to the basic radiobiology of carbon ion irradiation compared to that of conventional gamma-radiation. This includes the relative biological effectiveness, the oxygen effect, and the multicellular radioresistance. The second chapter is focused on our findings regarding clinical aspects of undesirable side effects of the two radiation types considered. These aspects refer to radiation-induced cell motility and to radiation-associated mucositis. The third chapter of this review links our own data to findings from the literature. For many years, radiobiological studies on heavy charged particles have remained sparse, but very recently, there is a tremendous increase in the number of reports on radiobiology of heavy ions, on their clinical use, as well as on a combination of their radiobiological and clinical aspects. Consequently, the intention of the third chapter of this review is by no means to give a comprehensive review of the literature on particle irradiation, but rather to present a selection of very recent reports that are closely related to the data presented here. The final paragraph of this review presents a brief resume of the article.

## Basic Radiobiology of Heavy Charged Particles

### RBE and OER Values for Carbon Ion Irradiation of Multicellular V79 Spheroids

Ever since the pioneering work of Robert Sutherland and colleagues (2), reviewed in Ref. (3), MCS represent classical 3D cell models in radiation research. Based on a sabbatical in Sutherland’s laboratory (4) and on the pioneer’s personal assistance as a Humboldt awardee at the University of Mainz (5), one of us set up a state-of-the-art spheroid laboratory at our research institute. Within the frame of a number of different research projects, we collected a large amount of data on 3D versus 2D growth characteristics, on 3D interaction between tumor and immune cells, or on tumor microenvironment with regard to hypoxia, hypoglycemia, acidosis, and other factors (6).

Occasionally, these data sparked the interest of scientists at the GSI in using our expertise with the spheroid technology for the exploration of heavy charged particle radiobiology. Spheroids from immortalized and tumorigenic V79 hamster cells have been frequently used in radiobiological investigations, and we decided to initiate our studies on heavy ion radiobiology with this spheroid type having an abundance of comparative data from X-ray experiments.

**Figure 1:**
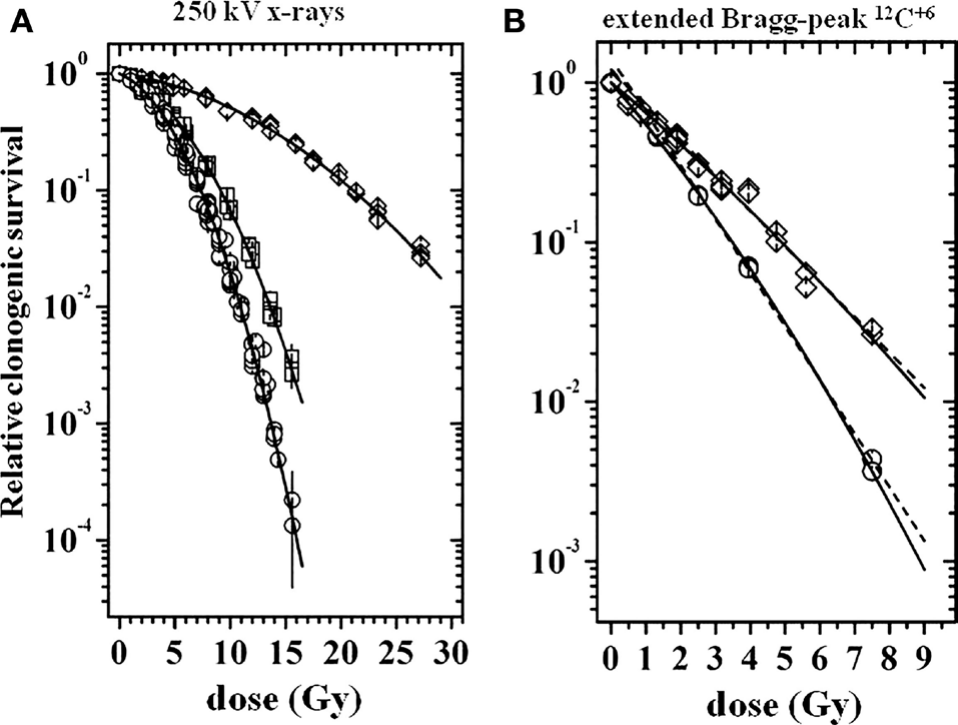
**Relative clonogenic survival of cells from V79 MCS as a function of dose [modified according to Ref. (7)]**. **(A)** Following X-ray irradiation at external pO_2_ values of 144 mmHg (circles), 35 mmHg (squares), and 0 mmHg (diamonds), **(B)** following ^12^C^6+^ irradiation in the extended Bragg peak at external pO_2_ values of 690 mmHg (circles) and 0 mmHg (diamonds); dose values represent X-ray equivalent dose.

Multicellular spheroids from V79 cells with 200 μm in diameter were irradiated with X-rays or carbon ^12^C^6+^ ions under elevated, ambient, or restricted oxygen supply conditions. From previous microelectrode measurements, the oxygen tension distribution within the MCS as a function of the external oxygen tension was known, which made it possible to exactly relate the local oxygen to the radiation effects. For reasons of simplicity, average numbers for the environmental oxygen tension (pO_2_) in mm Hg are given for characterizing the experimental conditions. Figure 1 shows clonogenic cell survival curves for V79 MCS irradiated with X-rays (Figure 1A) in environmental pO_2_ values of 144 mmHg (circles), 35 mmHg (squares), and 0 mmHg (diamonds) or irradiated with ^12^C^6+^ ions in the extended Bragg peak (Figure 1B) in environmental pO_2_ values of 690 mmHg (circles) and 0 mmHg (diamonds). It is obvious that (i) survival curves after particle irradiation are close to being linear with almost no shoulder compared to the X-ray data and (ii) the oxygen effect is much less pronounced with particle compared to photon irradiation. Survival curves of V79 single cells were almost identical with that of V79 spheroids with no indication of a multicellular resistance or contact effect (data not shown). Irradiation of V79 MCS with carbon ions in the plateau region (227 MeV/nucleon ^12^C^6+^) in the same oxygen atmospheres as used with X-ray treatment produced survival curves that were almost identical with those from photon irradiation (data not shown).

The survival curves of V79 MCS displayed in Figure 1 were fitted with the linear quadratic model. This was used for deriving reliable OER and relative biological effectiveness (RBE values). OER values at several survival levels *S* (=37, 10, 1, and 0.1%) were calculated as the ratio of doses to achieve a given survival under hypoxia compared to normoxia. Averages were derived from the individual values, which varied with *S* by around 5%. A corresponding procedure was used for the derivation of RBE, which was defined as the ratio of X-ray dose to that of particle radiation to reach a given *S*. These data are compiled in Table 1. Besides the very low OER value of 1.40 for particle irradiation, the RBE value of heavy charged particles is remarkably high at 4.31. Further details on the data evaluation were published earlier (7).

**Table 1:** **Oxygen enhancement ratio (OER) and relative biological effectiveness (RBE) values derived from the clonogenic survival curves shown in Figure 1 after irradiation in atmospheres with a pO_2_ of 690 mmHg or 145 mmHg (aerobic) or 0 mmHg (hypoxic)**.

Radiation type	OER	RBE	
		Aerobic	Hypoxic
X-rays	2.87	1.00	1.00
Ext. Bragg peak	1.40	2.11	4.31

**Figure 2:**
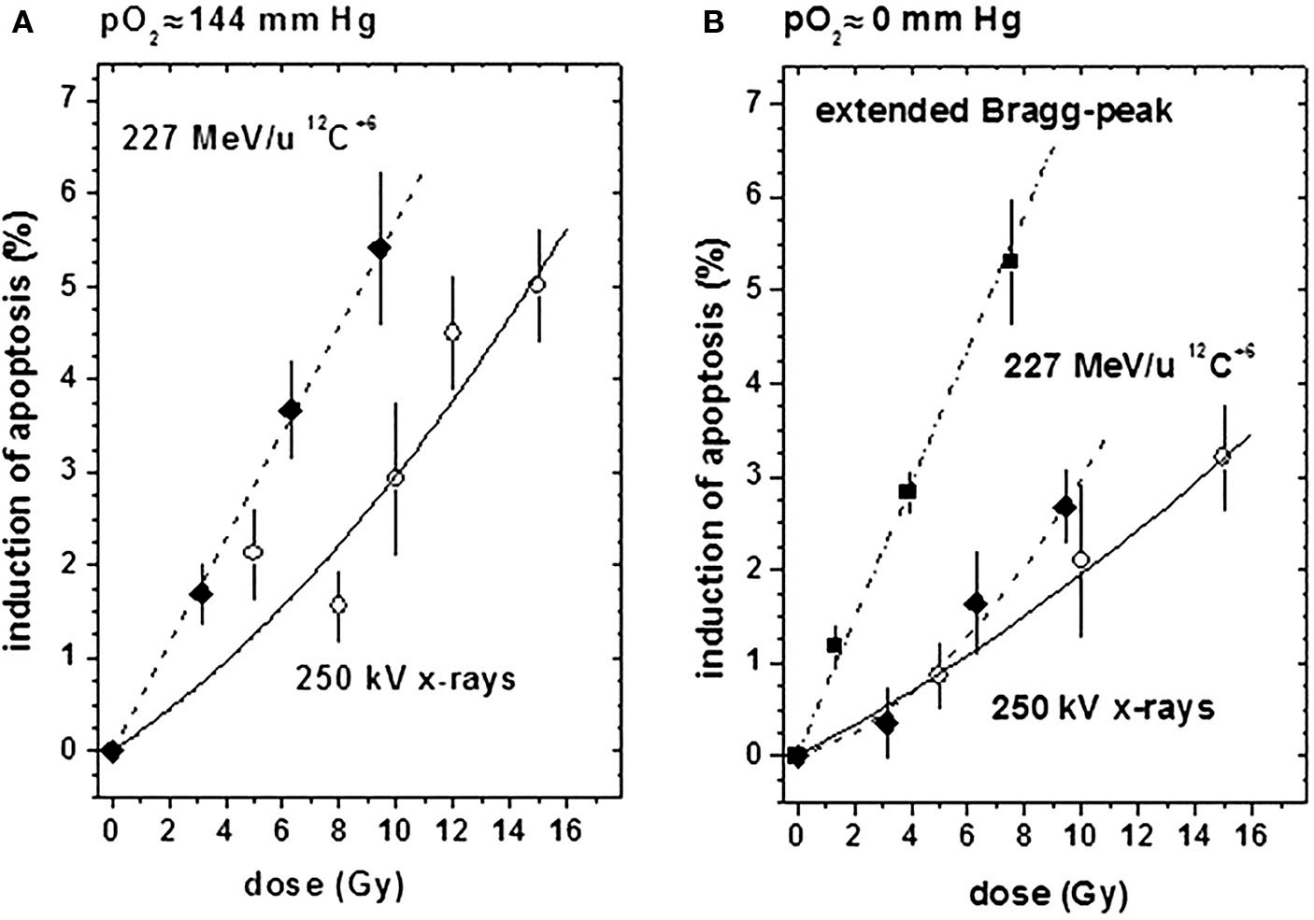
**Induction of apoptosis (relative to untreated controls) by X-ray (open circles) or ^12^C^6+^ irradiation in the plateau region (filled diamonds) or in the extended Bragg peak (filled squares) under ambient or hypoxic oxygen supply conditions [modified according to Ref. (7)]**. **(A)** At an external pO_2_ of 144 mm Hg and **(B)** at an external pO_2_ of 0 mm Hg.

Furthermore, the high effectiveness of heavy charged particles in the extended Bragg peak compared to conventional radiation was reflected by a massive, dose-dependent induction of apoptosis [quantified by the TUNEL assay (7)], as shown in Figure 2. Although a respective curve for the extended Bragg peak induction of apoptosis under ambient oxygen conditions could not be assessed in this set of experiments for technical reasons, explorative data were indicative of an absence of an oxygen effect with regard to apoptotic cell kill [for further details, see Ref. (7)]. All data obtained in this spheroid study clearly show that heavy charged particles are more efficient in sterilizing tumor cells than conventional irradiation even under hypoxic conditions.

### Unexpected Multicellular Radiosensitization in Human Colon Adenocarcinoma-Derived Multilayer Cells

**Figure 3:**
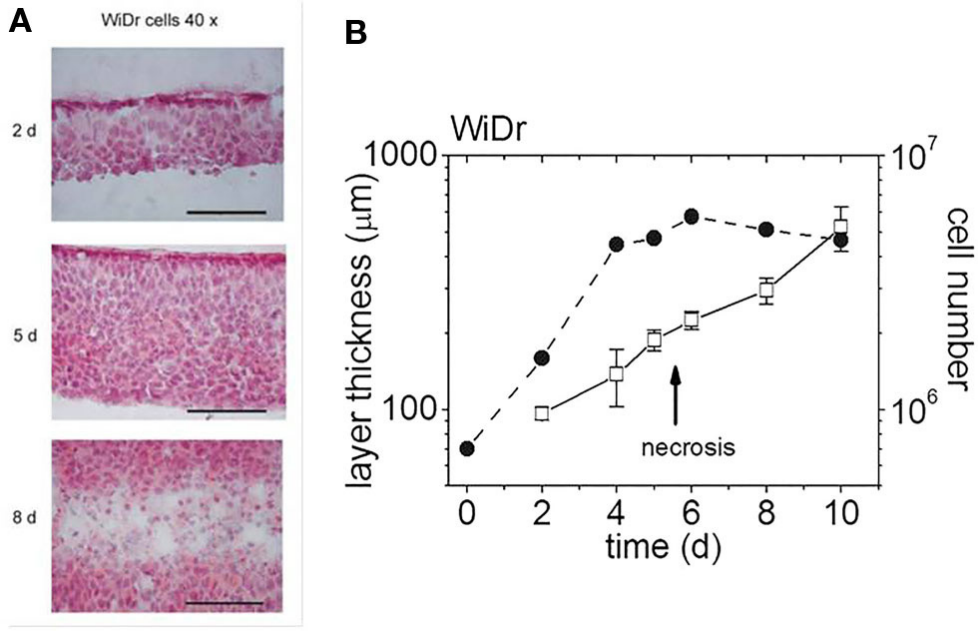
**Multilayers of WiDr cells at various days in culture [modified according to Ref. (7, 8)]**. **(A)** H&E-stained cryosections of multilayers on three different days (d) in culture (scale bar: 100 μm), **(B)** multilayer thickness and cell content as a function of days in culture (the arrow indicates the emergence of a central necrotic layer).

Planar cell multilayers in comparison with monolayer cultures of WiDr and SiHa human colon adenocarcinoma-derived cells were used for investigations on the role of cell cycle effects in the treatment with photon or particle irradiation. Development of a special cryostat sectioning technique made it possible to assess histology and growth characteristics of the planar 3D model (8). This is exemplified by Figure 3, with Figure 3A displaying cryostat sections that were cut perpendicular to the multilayer plane at three different times of growth for both cell lines considered. Figure 3B shows the multilayer thickness and viable cell content as a function of time in culture. Obviously, the total layer thickness is expanding continuously after the emergence and expansion of a central necrotic layer, whereas the total number of viable cells is stagnating. As such, planar cellular multilayers grow in a way, which is very similar to that of MCS despite the different diffusion geometries.

**Figure 4:**
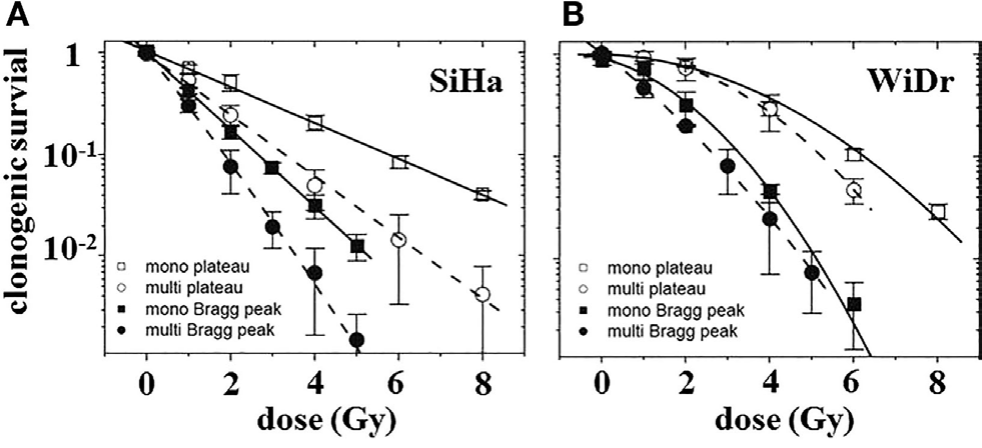
**Clonogenic survival of cells in monolayer (squares) or multilayer (circles) culture as a function of dose after carbon-ion irradiation in the plateau region (370 MeV/nucleon; open symbols) or in the extended Bragg peak [closed symbols; modified according to Ref. (8)]**. **(A)** SiHa cells (*n* = 4) and **(B)** WiDr cells (*n* = 2–3).

Unexpectedly, there was a multicellular radiosensitization of multi- versus monolayers under all treatments considered, as demonstrated by standardized clonogenic survival curves for ^12^C^6+^ ion irradiation in the plateau region and the Bragg peak (Figure 4). This is in contrast to the generally detected multicellular radioresistance. The phenomenon was attributable, at least in parts, to a difference in the proportion of cells in the G_0_/G_1_ phase between the two culture types used. Furthermore, Figure 4 illustrates cell line-dependent differences in the type of cell survival curves: whereas WiDr cells exhibit “classical” shoulder curves except for Bragg peak particle irradiation, SiHa cell survival curves are very close to linearity with all treatments considered. This difference may indicate a different extent of DNA repair in these two cell lines due to either a different intensity/quality of DNA damage or different repair capacities.

**Figure 5:**
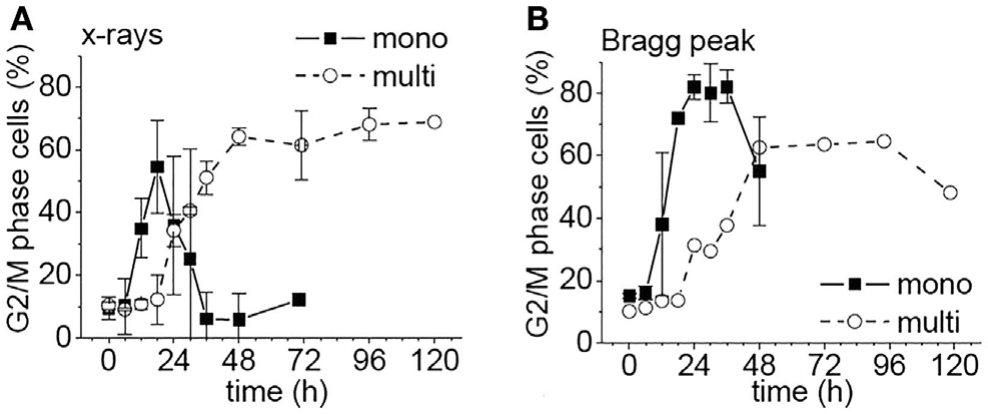
**Percentage of WiDr cells from mono- or multilayers in G_2_/M phase as a function of time after irradiation [modified according to Ref. (8)] with (A) X-rays and (B) carbon ions**.

Flow cytometric studies showed that X-rays induced a G_2_/M arrest, which was considerably prolonged in multi- compared to monolayers (Figure 5A). After Bragg peak irradiation of monolayers, the arrest time was increased compared to X-rays by 12–24 h, and more cells were arrested than with X-rays (Figures 5A,B). However, in multilayers, both radiation modalities lead to similar growth arrests (see Figures 5A,B).

In essence, our data obtained in the multilayer project contribute to accumulating results in the literature regarding differences in biological properties and molecular mechanisms between 2D and 3D culture systems. Under many aspects, 3D planar cellular multilayers and 3D spherical cellular aggregates share common properties and behave in similar ways. On the other hand, multilayers tend to show a small but reproducible multicellular radiosensitization, whereas most spheroids exhibit a multicellular resistance. It is worth noting that this effect is even more pronounced with heavy charged particles than with photons [for further details on planar multilayers, see Ref. (8)].

## Clinical Aspects of Potential Side Effects of Heavy Charged Particle Irradiation

### Differential Effects of Radiation on Cell Migration Depending on Radiation Type and Cell Line

When we initiated this project, there was an ongoing controversy within the science community about radiation-related modulation of tumor cell migration, mainly with regard to irradiation of glioblastomas (GBMs). The cellular motility of certain tumor cell lines is enhanced under *in vitro* conditions by sublethal doses of photon irradiation, as we and others have previously reported (9–11). In contrast, several other studies demonstrated that either similar sublethal or slightly higher doses impair GBM cell migration and invasion (12) or fail to modify these cell functions (13). The clarification of the respective controversy is clinically highly relevant: if therapeutic irradiation would enhance cell motility, tumor cells may leave the therapeutic field without receiving a cytotoxic dose and may thus be a source of recurrence. Some recent data were suggestive of heavy charged particle irradiation to consistently reduce the migratory potential of tumor cells, but the respective study was lacking parallel evaluation of radiation-induced cell killing (14).

Recent therapeutic strategies target the epidermal growth factor receptor (EGFR), which is overexpressed in about 40–50% of GBMs (15). There is evidence in the literature for such anti-EGFR therapeutics to improve the efficacy of conventional radiotherapy (16). EGFR is a member of the cell-surface receptor family ErbB and functions as an oncogene. Activation of EGFR by binding of its specific ligands, including epidermal growth factor (EGF), leads to dimerization of the receptor and subsequently to autophosphorylation of its tyrosine-kinase domain. Following activation, the EGFR kinase stimulates a number of cellular signaling cascades, such as the phosphatidylinositol-3-kinase (PI3K)/AKT or the mitogen-activated protein kinase (MAPK) pathway (17). Thereby, numerous cellular responses are precisely regulated, such as proliferation, cell survival, and cell migration. EGF-induced EGFR activation has been shown to promote tumor cell migration (18). In addition to auto- and paracrine stimulation, it remains to be clarified whether therapies, such as radiotherapy, induce EGFR activation and pro-proliferative signaling directly or indirectly *via* production of radicals. Before starting our research in this field, no data existed with respect to EGFR activation by heavy charged particle irradiation.

Considering this background information, we initiated investigations on the impact of carbon ion irradiation on GBM cell motility and EGFR-related cell signaling *in vitro*. Most cell migration data were generated in “classical” Boyden (or transwell) chamber assays. Core proteins and phospho-proteins were analyzed with Western Blotting.

**Figure 6:**
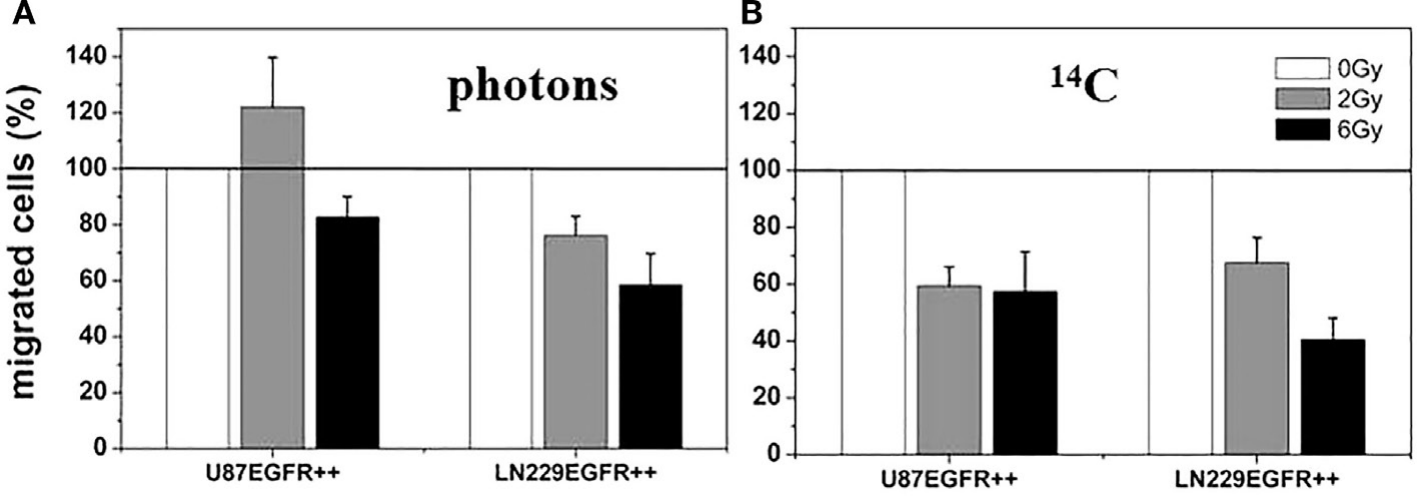
**Relative cell migration 24 h after photon and ^12^C heavy ion irradiation**. U87 EGFR^++^ and LN229 EGFR^++^ were irradiated with single doses of 2 and 6 Gy of photon radiation, respectively **(A)** and ^12^C heavy ions **(B)** and migrated cells were counted after Boyden Chamber assay. The relative mean numbers of migrated cells ± SD of at least three independent experiments are plotted with the migration of untreated cells set to 100%. All differences between treated and untreated cells are significant (*p* < 0.05, *t*-test). There was no significant change in cell viability under all conditions investigated (data not shown) [modified according to Ref. (19)].

**Figure 7:**
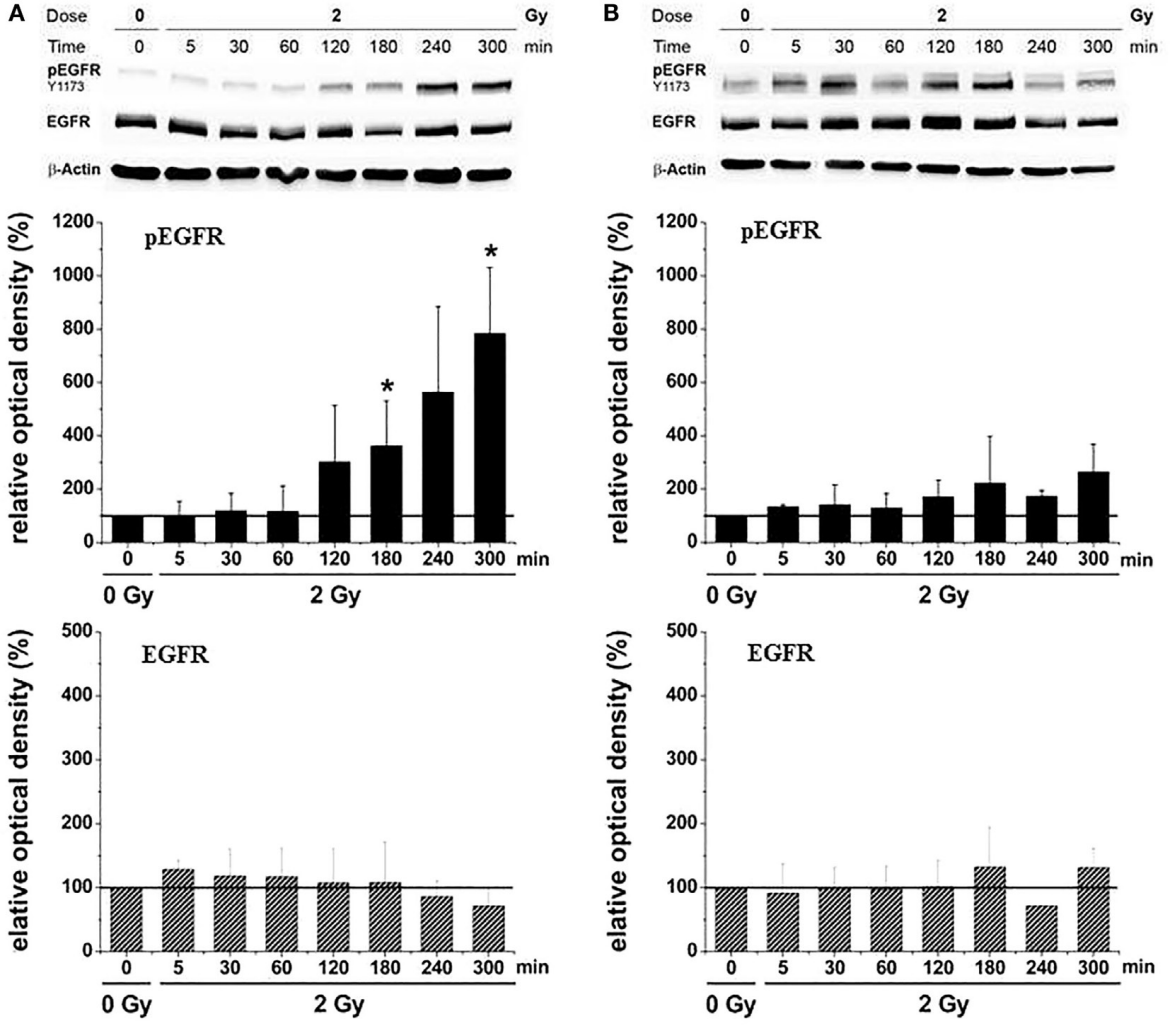
**Relative EGFR-phosphorylation (pEGFR) and total EGFR-protein (EGFR) in U87 glioma cells as a function of time after irradiation with 2 Gy [modified according to Ref. (19)] applying (A) X-rays and (B) carbon ions**.

**Figure 8:**
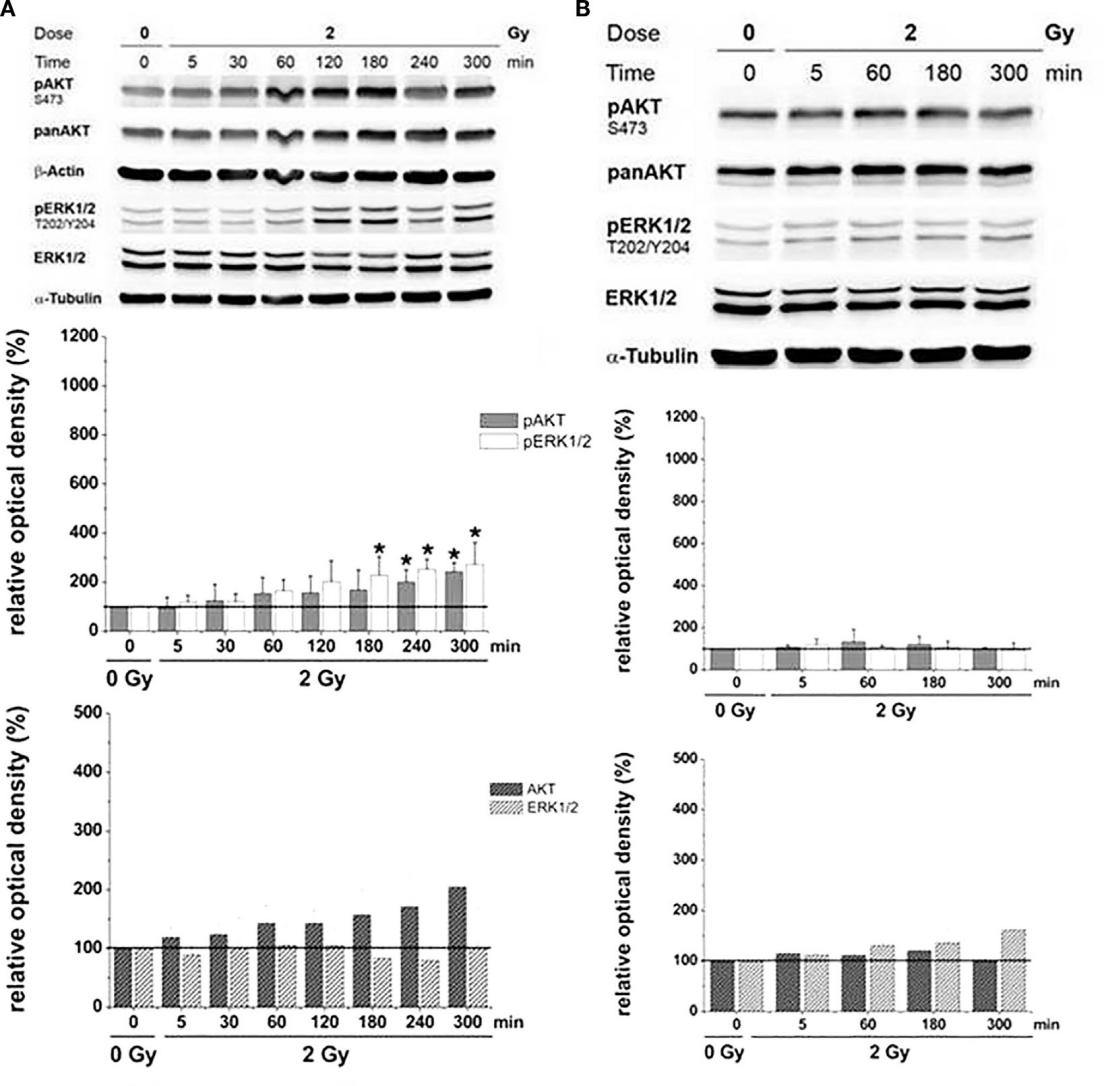
**Relative AKT/ERK-phosphorylation (pAKT/pERK) and total AKT/ERK-protein (AKT/ERK) in U87 glioma cells as a function of time after irradiation with 2 Gy [modified according to Ref. (19)] applying (A) X-rays and (B) carbon ions**.

Investigations on U87 and LN229 glioma cells (with overexpression of EGFR^++^) showed that the migratory response of cancer cells to radiation is dependent on radiation dose, as well as on cell and radiation type. Clinically relevant doses (2 or 3 Gy) of X-rays induced a small, but consistent and significant, increase in migratory activity of U87, but not of LN229, as illustrated in Figure 6A. ^12^C^6+^ ion treatment was associated with a dose-dependent decrease in migration with all cell lines and under all conditions investigated (Figure 6B). The radiation-induced loss of cell motility was correlated, in most cases, with corresponding changes in β_1_ integrin expression (9, 14). The photon-induced increase in cell migration in U87 glioma cells was paralleled by an elevated phosphorylation status of the EGFR and AKT–ERK1/2 pathway (see Figures 7 and 8). Such a hyperphosphorylation did not occur during ^12^C^6+^ irradiation under all conditions registered (see Figures 7 and 8). Using a 3D collagen type I invasion and migration model, glioma cell migration remained unaffected by irradiation with either photon or particles, despite the induction of massive gene toxicity as determined by the γH2AX technique (13).

On the one hand, with a few exceptions, our *in vitro* findings on the interrelationship between irradiation and tumor cell migration are in accordance with data from the literature (14, 20, 21). On the other hand, *in vivo* studies are warranted for the evaluation of the clinical significance of this issue.

Four major conclusions were derived from the *in vitro* migration studies on glioma cells: (i) the impact of radiation on glioma cell migration depends on the migration assay used with both X-rays and carbon ions; (ii) under certain conditions and in a few glioma cell lines, clinically relevant doses of photons but not particles consistently increases cell migration; (iii) under a wide spectrum of conditions, glioma cell migration *in vitro* was either unaffected or reduced by ^12^C^6+^ irradiation; and (iv) this differential between photon and particle irradiation may contribute to a higher efficiency of a local carbon ion treatment compared to X-rays with regard to tumor recurrence.

### Studies on Early Events in Radiation-Induced Mucositis Using Organotypic Cultures of the Oral Mucosa Including Immune Cells

Oral mucositis is a frequent complication of standardized radiotherapy in the clinic. There is an abundance of literature regarding preclinical and clinical research in this field, as reviewed recently, among others, by Mallick and colleagues (22). Much less is known, in this regard, about the side effects of particle irradiation, although the induction of mucositis by carbon ions has been clearly documented in patients already in 2002 (23). Although the number of centers for treatment with heavy charged particle is still undesirably low, the successful application of this technology in radiation oncology for the past two decades confers clinical relevance to particle treatment-associated mucositis (24).

A relatively novel aspect of radiation-associated mucositis results from the ambitious plans of several space agencies, in particular of the NASA and the ESA, for manned missions to the Mars. During such a mission which would last around 3 years, astronauts would be chronically exposed to cosmic radiation due to the absence of a protecting magnetic field. Space radiation consists of protons (87%), α-particles (12%), and heavy ions (1%) in solar particle events and galactic cosmic rays (25). In particular, highly ionizing heavy ions can be hardly shielded exposing the crew members to a serious medical safety risk (26), since the probability of getting a hit by heavy charged particles increases with time in space. It is obvious that the occurrence of oral or intestinal mucositis during a prolonged space flight would lead to hazardous situations.

Oral mucositis as a result of X-ray exposure has been studied in numerous animal models, the advantages and limitations of which have been reviewed recently by Viet and co-workers (27). One major cutback of animal models that has been reported earlier is their unsuitability for the assessment of early molecular and pathophysiological events following irradiation (28).

Based on this background knowledge, we initiated a project, which was supported mainly by the GSI Darmstadt and the ESA, with investigations on early inflammatory events induced by heavy charged particle irradiation in organotypic cultures of the human oral mucosa. We re-activated a 3D culture model, previously established in our laboratory (29). The artificial mucosa, which was cultured at the liquid–gas interface, consisted of immortalized human gingival keratinocytes (IHGK) and immortalized human dermal fibroblasts (HH4ded), grown with or without PBMCs. The organotypic mucosa culture exhibited many features of the human oral mucosa, such as the formation of a basal membrane, a papillary shape of the epithelium-connective tissue boundary, or the differentiation status of the keratinocytes with regard to expression of keratins. A special technology was designed making it possible to irradiate the 3D cultures in the extended Bragg peak of the heavy ion beam including an appropriate dosimetry. Further details are described in Ref. (30).

**Figure 9:**
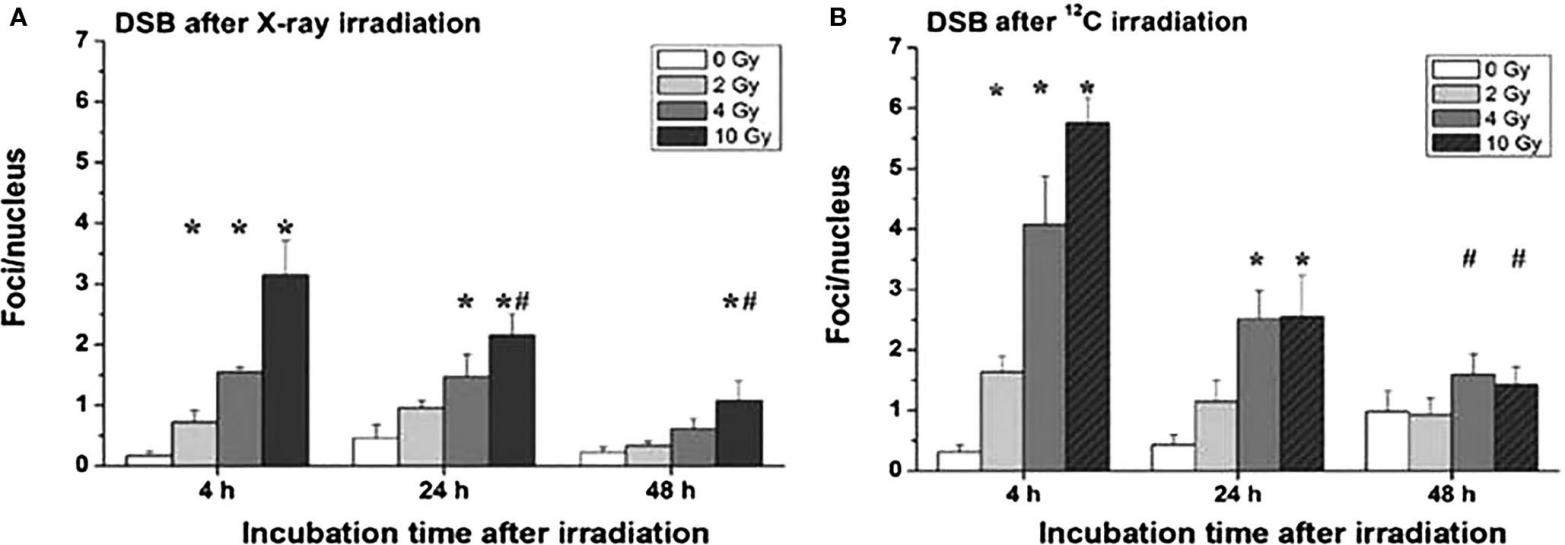
**Double-strand breaks (DSB) in organotypic cultures of oral mucosa, determined by evaluation of γH2AX stainings in immune fluorescence microscopy, as a function of radiation dose and time after radiation [modified according to Ref. (19)] applying (A) X-rays and (B) carbon ions**.

Comparing the gene toxicity of X-rays with that of particles using the γH2AX technique, the superior effectiveness of heavy ions was confirmed by a roughly twofold higher number of foci per nucleus 4 and 48 h after treatment. This is shown in Figure 9 for X-rays (Figure 9A) and ^12^C^6+^ irradiation (Figure 9B).

**Figure 10:**
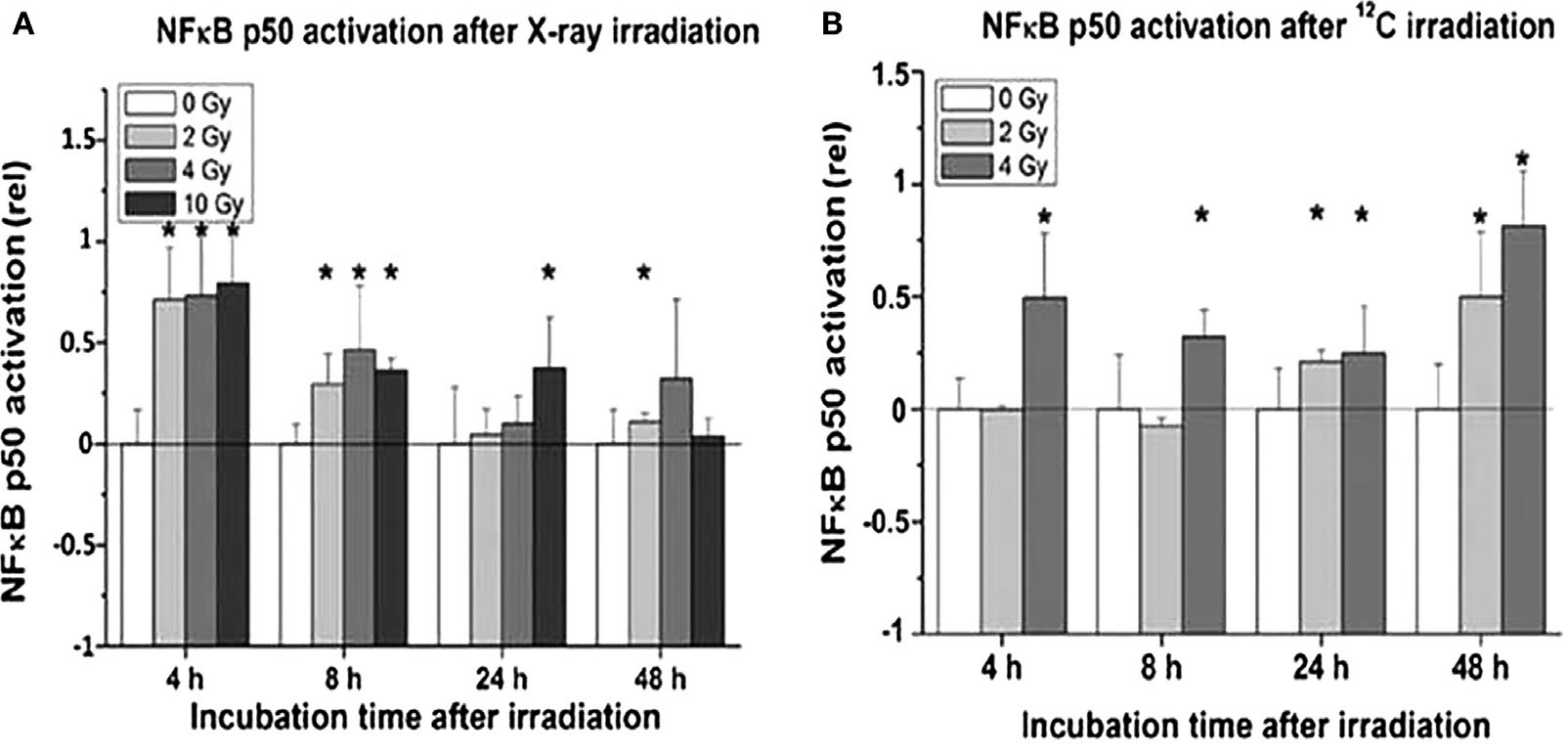
**Activation of NFκB in organotypic cultures of oral mucosa, determined by evaluation of nuclear translocation of NFκB p50 stainings in immune fluorescence confocal microscopy, as a function of radiation dose and time after radiation [modified according to Ref. (19)] applying (A) X-rays and (B) carbon ions**.

**Figure 11:**
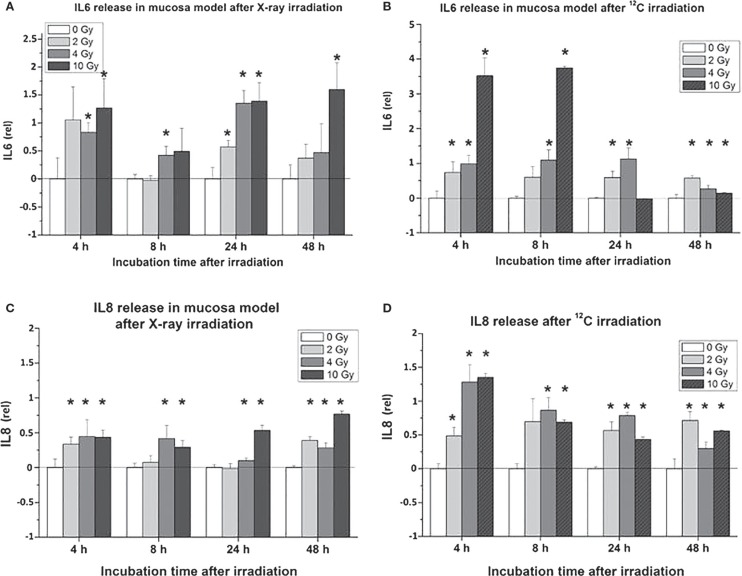
**Cytokine release [IL6: (A,B); IL8: (C,D)] in organotypic cultures of oral mucosa, determined by commercial ELISA test, as a function of radiation dose and time after radiation [modified according to Ref. (19)] applying (A,C) X-rays and (B,D) carbon ions**.

**Figure 12:**
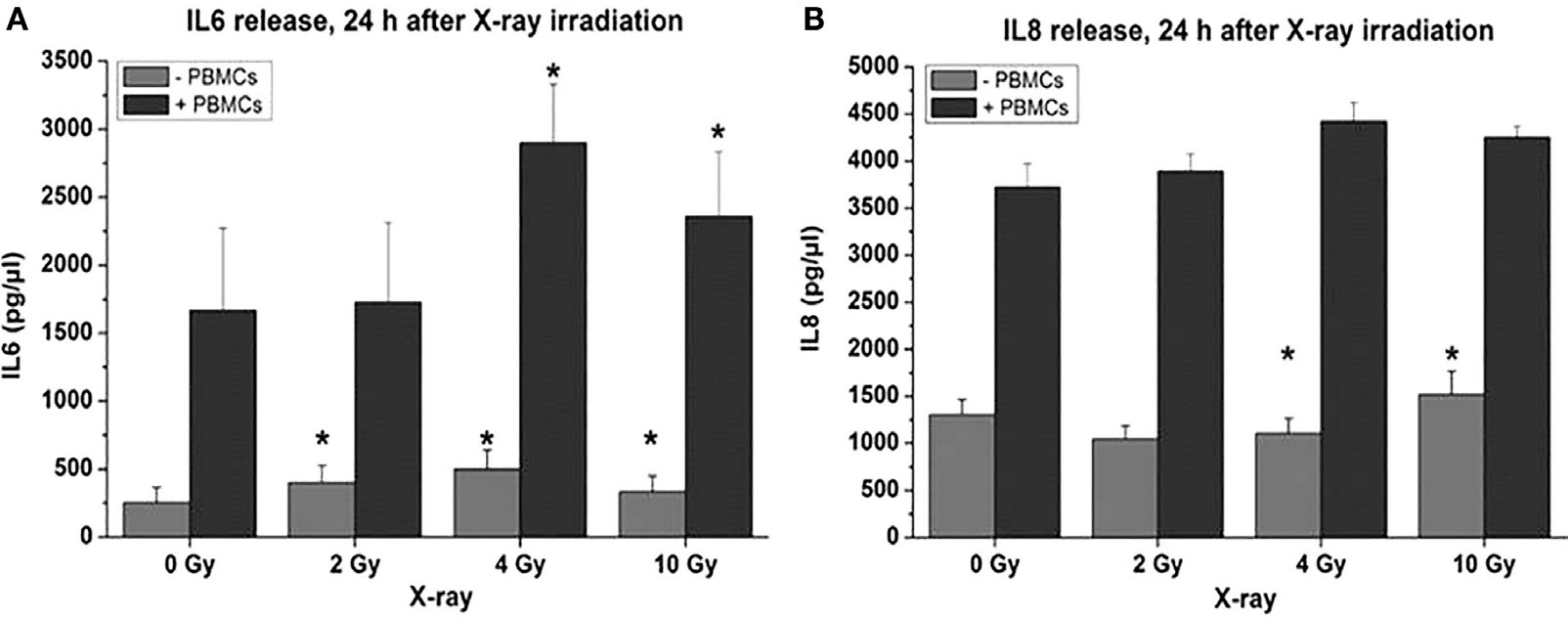
**Cytokine release following X-ray treatment of organotypic cultures of oral mucosa, determined by commercial ELISA test, as a function of radiation dose and time after radiation with or without coculturing of peripheral blood mononuclear cells [PBMCs; modified according to Ref. (19)]**. **(A)** IL6 and **(B)** IL8.

Proinflammatory signs were quantitatively similar for both treatment modalities. For example, confocal microscopy made it possible to quantify the activation of NFκB by the assessment of the nuclear location of NFκB p50. The corresponding results are depicted in Figures 10A,B for photons and particles, respectively. The release rates of the proinflammatory cytokines IL6 and IL8 from the organotypic cultures into the culture medium was registered using commercial ELISA assays [further details in Ref. (30)]. Figure 11 demonstrates a consistent and significant elevation of the release of both cytokines upon irradiation for both photons (Figures 11A,B) and particles (Figures 11C,D), albeit in the absence of a consistent dose dependency. Figures 12A,B illustrate for X-rays and carbon ions, respectively, that the addition of PBMC increases the radiation-induced release of IL6 and IL8 by factors of 2–3.

## Discussion and Resume

### Data from the Literature

The very recent literature on heavy charged particle research clearly emphasizes the advantages of particle versus X-ray irradiation in a meanwhile broad spectrum of tumor entities as shown in a large number of patients mainly in Japan (more than 8,000 patients) and Germany (31, 32). Whereas different ions, such as carbon, helium, or protons (33), may be used in different treatment scenarios, carbon and helium appear to be superior to protons in the majority of cases (34). One review lately points out the importance of combining radiobiological and clinical research with carbon ion therapy (35–37). There is a common optimism among these authors with regard to further spread of charged particle treatment facilities world-wide (31, 32, 34, 38). Besides these clinical aspects, the already-mentioned significance of charged particle radiobiology for long-term exposition to space radiation during extended space flights has been detailed explicitly by an international consortium of experts in a recent article (39).

Several actual reports on cell and animal studies using carbon ions present RBE values, which are in a fairly good agreement with our data from multicell spheroid studies (35, 40, 41). At the same time, data are presented that show a multitude of parameters to impact on RBE values, such as radiation dose, linear energy transfer (LET), and the model used for the derivation of RBE (35, 37). Taking this into account, the derivation of RBE values for clinical dosimetry is still a matter of debate (37). In a recent simulation study on hypoxia in clinical tumors treated with carbon ions, OER were found that corresponded well to our data from MCS (42). The authors also point to the existence of a non-negligible oxygen effect that can influence the outcome of carbon ion therapy at low LET in the spread-out Bragg peak. A recent investigation using a carbon ion beam or X-rays for irradiating neuro-spheres of human GBM cells documents the occurrence of a multicellular resistance that was much less pronounced – but still detectable – with particle radiation compared to photons (43). As many studies with X-rays before, this investigation demonstrates a multicellular radioresistance, eventually termed contact effect, to exist for carbon ions as well. This is in contrast to the multicellular radiosensitization, which we have shown for charged particle irradiation of planar tumor cell multilayers compared to corresponding single cells [(8); see above].

There are two recent reports confirming our observation on charged particle effects on cell migration and related signaling pathways. Simon et al. (44) were able to show that migration of meningioma cells was promoted by photon but not by carbon ion irradiation. Our results on an increased cell migration associated with an elevated phosphorylation status of the EGFR and AKT–ERK1/2 pathway following X-ray but not carbon irradiation was partially confirmed by corresponding findings of Jin and colleagues (45). These data are in accordance with previous observations regarding the inhibitory influence of heavy charged particle irradiation on tumor cell migration and formation of metastasis (21, 46). Recent reviews, such as the article by Fujita (47), mirror a still ongoing and partially controversial debate with regard to the impact of radiation on cancer cell motility, invasiveness, and metastatic potential. Interestingly, a recent experimental study using carbon ion irradiation in a rat prostate carcinoma has demonstrated an increase in the metastatic rate upon treatment (48).

As a brief resume, the experimental therapeutics part of the data compiled clearly demonstrates the efficiency of ^12^C^6+^ irradiation to be consistently higher than that of conventional X-rays; this is mirrored by RBE values for particles versus photons of >1.0 and up to 4.3 under hypoxic conditions. Since hypoxia occurs in 50–60% of all human solid tumors (49), it is of high clinical relevance that ^12^C^6+^ irradiation is much more efficient than conventional radiation under these conditions. Whereas OER values are close to 3 for X-rays, an OER value of 1.4 was derived for carbon ions. Here, multicellular tumor spheroids proved themselves as useful models for quantitative studies on the radiobiology of heavy charged particles. In the experimental inflammation part of the data compilation, organotypic cultures of the oral mucosa were shown to be useful for investigations on immediate and early inflammatory events, i.e., within a few hours up to 2 days following ^12^C^6+^ radiation treatment. Besides the quantification of gene toxicity and proinflammatory cytokine release, the consistent, immediate, and early, as well as dose-dependent activation of NFκB by ^12^C^6+^ irradiation of oral mucosa cultures is one of the core results of this part of the studies.

## Author Contributions

Both the authors listed have made substantial, direct, and intellectual contribution to the work and approved it for publication.

## Conflict of Interest Statement

The authors declare that the research was conducted in the absence of any commercial or financial relationships that could be construed as a potential conflict of interest.
